# Multiple pituitary hormone deficiency: beware of combined hormones deficiency

**DOI:** 10.1186/1687-9856-2013-S1-P198

**Published:** 2013-10-03

**Authors:** Nur Rochmah, Muhammad Faizi, Achmad Yuniari, Netty Harjantien

**Affiliations:** 1 Department of Child Health,School of Medicine, Airlangga University-Dr. Soetomo Hospital, Surabaya, Indonesia

**Keywords:** growth hormone deficiency, hypogonadotropic hypogonadism, multiple pituitary hormone deficiency, diagnosis, treatment

## 

Multiple Pituitary Hormone Deficiency (MPHD) is an endocrine disorder due to combination of pituitary hormones deficiencies. Clinical manifestations vary due to the combination of individual hormone deficiencies. The diagnosis is established based on history, signs and symptoms, hormonal and radiological examination. MPHD should be managed by hormones replacement according hormone abnormalities. The objective is to present a case of multiple pituitary hormones deficiency in a child, focusing in diagnosis and management.

A 14 years-old boy, come with main complaint of short stature and delayed puberty. The height was below the 3^rd^ percentile for age. Patient also suffered from micropenis. Tanner stage was prepuberta. Laboratories showed peak stimulated growth hormone concentration test was below 10 ng/mL, low levels of IGF 1 and IGF-BP3; FT4: 5.99ng/dl (normal:4.6-11),TSH: 3.08 IU/mL (normal:0.4-7.0); non stimulated LH level: 0.23 mIU/µL (cut-off limit prepubertal-pubertal level was 0.6 IU/L), FSH: 1.6 mIU/µL; testosrone: <15 ng/dL (normal basal testosterone: 19 ng/L). Working diagnosis was GH deficiency and hypogonadotrophic hypogonadism. This patient treated with growth hormone and testosterone. The height increased 10 cm during 10 months growth hormone treatment and the penile length increased became normal range. This paper reported growth hormone deficiencies accompanied by hypogonadotrophic hypogonadism. Facing patient with pituitary hormone deficiency must be aware of the abnormality of other pituitary hormone.

